# Identification, Detection, and Management of Soft Rot Disease of Ginger in the Eastern Himalayan Region of India

**DOI:** 10.3390/pathogens14060544

**Published:** 2025-05-29

**Authors:** Utpal Dey, Shatabhisa Sarkar, Durga Prasad Awasthi, Mukesh Sehgal, Ravinder Kumar, Biman De, Nayan K. Adhikary, Abhijit Debnath, Rahul Kumar Tiwari, Milan Kumar Lal, Subhash Chander, Ph. Ranjit Sharma, Amulya Kumar Mohanty

**Affiliations:** 1 Krishi Vigyan Kendra, Sepahijala, CAU(I), Latiacherra 799102, Tripura, India; utpaldey86@gmail.com (U.D.); shatabhisasarkar@gmail.com (S.S.); 2 College of Agriculture, Tripura, Lembucherra 799210, Tripura, India; biman_de@rediffmail.com; 3 ICAR—National Research Centre for Integrated Pest Management, Mehrauli 110068, Delhi, India; msehgalncipm@aol.com (M.S.); subhash.chander6@icar.gov.in (S.C.); 4 ICAR—Indian Agricultural Research Institute, New Delhi 110012, India; 5 School of Agricultural Sciences, Nagaland University, Medziphema 797106, Nagaland, India; nayan.bckv@gmail.com; 6 Krishi Vigyan Kendra, Dhalai, Salema 799278, Tripura, India; abhijitdebnathhorticulture@gmail.com; 7 ICAR—Indian Institute of Sugarcane Research, Lucknow 226002, Uttar Pradesh, India; rahultiwari226@gmail.com; 8 ICAR—National Rice Research Institute, Cuttack 753006, Odisha, India; milan2925@gmail.com; 9 Directorate of Extension Education, Central Agricultural University, Imphal 795004, Manipur, India; ranjitsharmaph@gmail.com; 10 ICAR—Agricultural Technology Application Research Institute, Zone VII, Umiam 793103, Meghalaya, India; dramulyakumar@gmail.com

**Keywords:** biocontrol agents, management, *Pythium aphanidermatum*, soft rot, *Zingiber officinale*

## Abstract

Ginger is an important spice crop in the north-eastern region of India. Rhizome rot, also called soft rot, is one of the most devastating diseases found in ginger that causes yield losses of up to 100% under favourable conditions. Initially, the disease symptoms appear as a light yellowing of the leaf tips that gradually spreads down to the leaf blade of lower leaves and the leaf sheath along the margin. Under favourable environmental conditions, the disease spreads rapidly, potentially causing significant crop damage. The pathogen can infect at any stage of crop growth, and under favourable environmental conditions, the disease spreads rapidly, failing the crop. Current research emphasises mitigating the losses caused by the devastating disease by using management strategies and biocontrol agents (BCAs). Results revealed that the average highest percent rhizome germination, lowest mean disease incidence, lowest mean disease severity index, lowest coefficient of disease index value, highest rhizome yield and benefit–cost ratio were recorded with *Trichoderma harzianum* (10 g/kg of rhizomes) + soil application of *T. harzianum*-enriched well-decomposed farm yard manure (3 kg of *T. harzianum* mixed with 100 kg FYM at 10–15 days before sowing) + soil drenching with *T. harzianum* at the rate 10 kg/ha, compared to the untreated control. Furthermore, soil chemical properties such as pH, electrical conductivity, soil organic carbon, total available nitrogen, total available phosphorus, and total available potassium play critical roles in rhizome rot disease severity. BCAs can suppress the phytopathogenic fungi and modulate different functions in plants.

## 1. Introduction

Ginger (*Zingiber officinale* Rosc L.) belongs to the family Zingiberaceae and is an important commercial spice crop grown in many different states of India due to its aromatic rhizomes [1,2]. It has medicinal properties and can also be used as a spice for culinary purposes. India is the world’s largest ginger producer, producing 1.84 million tons from 0.17 million hectares [3], accounting for about 35 percent of total production [4]. Ginger is commercially grown in the North Eastern region of India, including the states of Assam, Arunachal Pradesh, Meghalaya, Manipur, Mizoram, Nagaland, Sikkim, and Tripura. The Eastern region produces 511.72 thousand tonnes [5] of ginger, accounting for about 23% of total production. The average yield of ginger in the eastern region is about 6.78 t/ha.

The ginger crop is affected by many pathogens, viz., fungi, bacteria [6], nematodes [2] and viruses [7]. Among the fungal diseases, rhizome rot, commonly known as soft rot, caused by *Pythium aphanidermatum* (Edson) Fitzp., is one of the limiting factors for the production of healthy rhizomes. The decrease in ginger rhizome yield due to soft rot disease was reported to be up to 70% in different parts of India [8] with crop losses potentially ranging from 50% to 90% under favourable conditions. Initial symptoms appear as a light yellowing of the leaf tips, which gradually spreads down to the leaf blade of lower leaves and the leaf sheath along the margin [9]. The pathogen may affect any crop growth stage, and the affected region becomes water-soaked. Generally, the infection starts at the collar region of pseudostems. However, the infection may also take place through the roots. Water-soaked lesions appear on the developing rhizomes near the base of the affected shoots and may be easily pulled out from the soil. Warm and humid weather conditions favour the disease. Under favourable environmental conditions, the disease spreads rapidly [10] and results in the complete failure of the crop [11]. The pathogen is soil-borne and spreads fast, so its management is difficult.

Beneficial microbes have tremendous potential for managing crop diseases [12,13]. These microbes produce antimicrobial natural compounds and secondary metabolites [14,15]. The antimicrobial natural compounds have a low molecular weight and are structurally varied [16,17]. *Trichoderma harzianum* produces peptaibols, polyketides, pyrones, terpenes, and non-ribosomal peptides [18]. *Pseudomonas fluorescens* produces 2,4-diacetyl phloroglucinol (DAPG), phenazine 1-carboxylic acid, siderophore, phenazines pyoluteorin, pyrrolnitrin fluorescein, pyocyanin and hydrogen cyanide, indole acetic acid (IAA), and hydrolytic enzymes [19,20]. The natural compound stimulates the plant defence mechanisms and provides broad-spectrum resistance to protect crop plants from phytopathogens [21]. Furthermore, beneficial microbes play a significant role in reducing the presence and concentration of plant pathogens, thereby increasing eco-friendly and sustainable production. The present study aimed to identify and confirm the disease’s causative agent and evaluate the performance of economically viable and ecologically safe fungicides against the soft rot disease of ginger under field conditions.

## 2. Materials and Methods

### 2.1. Description of the Study Area

The study was carried out during 2020 and 2021 at Chesrimai, Charilam block, Sepahijala district, Tripura, located approximately 8.9 km from Bishalgarh and 31 km from Agartala (capital of Tripura state), at 23.3610° N latitude and 91.1558° E longitude under the Humid Eastern Himalayan Region. The study area lies at an altitude of about 89 m.

### 2.2. Planting Materials

Locally grown varieties of ginger, which were on an average 3 cm long, 25–28 gm in weight, having 2 viable buds, are selected for sowing purposes.

### 2.3. Weather

**Figure 1:**
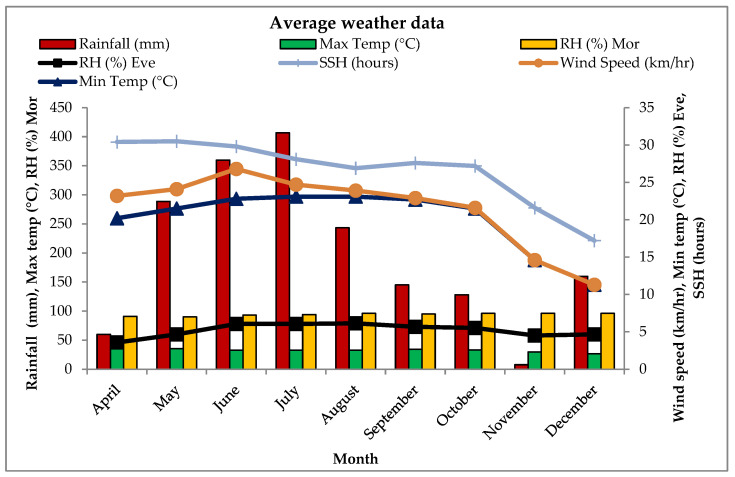
Average weather data for 2020 and 2021 for relative humidity evening (RH Eve), relative humidity morning (RH Mor), sunshine hours (SSH), maximum temperature (Max temp), and minimum temperature (Min temp).

The Sepahijala district, Tripura, has a warm and humid sub-tropical climate and climatic zones of the Humid Eastern Himalayan Region. The average minimum and maximum temperatures range from 11.0 to 23.5 °C and 26.3–34.7 °C, whereas average morning relative humidity and evening relative humidity range from 91 to 97% and 52.5–80.5%, respectively. The average annual rainfall is 215.6 mm during cropping (Figure 1).

### 2.4. Soil Chemical Properties

The soil samples were taken at 0–30 cm depths from the experimental site using an auger with a 5 cm internal diameter before establishing this experiment in April 2020 and after harvesting the ginger crop during 2020 and 2021. The soil samples were collected from an experimental site, one per replication and treatment, and distributed uniformly along a zigzag transects [22]. The composite sample was drawn after scrupulously mixing the samples. Soil samples were kept for shade drying, and after drying, the soil fraction passing through 30-micron sieves was collected and kept in plastic bags for chemical analyses. Soil pH, electrical conductivity (EC), soil organic carbon (SOC), total available nitrogen (N). phosphorus (P), potassium (K), and content of micronutrients *viz*., sulphur (S), manganese (Mn), boron (B), iron (Fe), copper (Cu), and zinc (Zn) was determined using Pusa Soil Test and Fertilizer Recommendation (STFR) metre developed by the Indian Agricultural Research Institute (IARI), New Delhi. The initial soil parameters of the experimental site are presented in Table 1.

### 2.5. Experimental Design and Treatments

The ginger rhizomes were planted in a plot size of 3 m × 1.2 m at a spacing of 30 cm × 30 cm with interspacing of 50 cm between beds, which were replicated thrice in a randomised block design in a participatory mode in the farmer’s field. Rhizomes were planted in the second week of April, and the recommended dose of fertiliser (75 kg N, 50 kg P_2_O_5_ and 50 kg K_2_O per ha) [23] were applied through well-decomposed Farm Yard Manure (FYM) at the rate of 10 t/ha and vermicompost at the rate 3.8 t/ha. Full doses of FYM and half doses of vermicompost at the rate of 1.9 t/ha were applied at the time of last ploughing, and rest vermicompost at the rate of 1.9 t/ha was applied at the time of earthing up 45 days after planting (DAP). Two-hand weeding was performed at 45 and 90 DAP. Different treatments were evaluated to determine fungicides’ efficacy against soft rot disease under field conditions. The various rhizome treatment details were T1, *T. harzianum* at the rate 10 g/kg of rhizomes followed by air drying for 24 h at a normal room temperature; T2, *P. fluorescens* at the rate 10 g/kg of rhizomes followed by air drying for 24 h at a normal room temperature; T3, copper oxychloride (COC) at the rate 0.3% (3 g/L) for 30 min and air dried before sowing; T4, *P. fluorescens* at the rate 10 g/kg of rhizomes + soil application of *P. fluorescens* enriched FYM (3 kg of *P. fluorescens* mixed with 100 kg FYM at 10–15 days before sowing) + soil drenching with *P. fluorescens* at the rate 10 kg/ha; T5, *T. harzianum* (10 g/kg of rhizomes) + soil application of *T. harzianum*-enriched well-decomposed farm yard manure (3 kg of *T. harzianum* mixed with 100 kg FYM at 10–15 days before sowing) + soil drenching with *T. harzianum* at the rate 10 kg/ha; T6, COC at the rate 3 g/L and streptomycin sulphate 90% + tetracycline hydrochloride 10% WP at the rate 0.2 g/L for 45 min followed by shade drying and soil drenching twice with COC at the rate 3 g/L at 60 and 90 days after planting (standard check); T7, control. Talc-based formulations of *T. harzianum* (2 × 10^6^ CFU/gm)/*P. fluorescens* (2 × 10^8^ CFU/gm) were mixed with 100 kg of FYM and incubated for 21 days before application to the main field to enhance growth and increase the antagonist population. In the control treatment, an equal amount of plain water was sprayed.

### 2.6. Evaluation of Parameters

The rhizome germination was calculated using the following formula [24]:

(1) $$Rhizome\;germination\;(\%)=\frac{Rhizomes\;germinated}{Total\;rhizomes\;planted}\times 100$$

The percentage disease incidence of the disease was calculated using the following formula [25].

(2) $$Percent\;disease\;incidence=\frac{Number\;of\;plant\;infected}{Total\;number\;of\;plants\;examined}\times 100$$

Based on the numerical rating/scores observed, the percent disease index (PDI) was calculated by applying the formula [26] as given below:

(3) $$PDI=\frac{Summation\;of\;numerical\;ratings}{No\;leaves/plants\;observed\times maximum\;rating}\times 100$$

Further, percent disease control (PDC) was obtained by applying the following formula:

(4) $$PDC=\frac{PDI\;in\;control\;plot-PDI\;in\;treatment\;plot}{PDI\;in\;the\;control\;plot}\times 100$$

The coefficient of disease index (CODEX) was also calculated [25].

### 2.7. Isolation and Characterisation of Pathogenic Fungi

Eight symptomatic samples were collected from the field in polypropylene bags and brought to the laboratory. Samples were washed in sterile distilled water to remove adhering soils. The pathogen was isolated in Potato Dextrose Agar (PDA) and Oat Meal Agar (OMA) media under aseptic conditions. A sterilised scalpel cut small bits of diseased parts and half-diseased and half-healthy tissues. Surface sterilisation of bits was performed by dipping them in 1% sodium hypochlorite solution for 10 to 15 s. Subsequently, samples were washed thoroughly 2–3 times in sterilised distilled water. The washed bits were transferred to PDA Petri plates under a laminar airflow. The inoculated Petri plates were placed in the Biochemical Oxygen Demand (BOD) Incubator at 28 ± 1 °C to isolate the pathogenic organism. The hyphal tip method on PDA Petri plates further purified the isolated organism. The isolated organism was further identified by studying its morphological and molecular characteristics. Further, the isolated organism (R. G. 1) was deposited to the National Fungal Culture Collection at Agharkar Research Institute, Pune, India, for identification.

### 2.8. Molecular Identification

Genomic DNA was extracted using pure colony growing on a PDA Petri plate and incubated for a week using a simple and rapid DNA extraction protocol and FastPrep^®^24 tissue homogenizer (MP Biomedicals GmbH, Eschwege, Germany) [27]. The internal transcribed spacer (ITS) region of rDNA was amplified using fungal universal primers ITS-5 and ITS-4 [28] with the initial denaturation at 94 °C for 5 min, 35 cycles of 1 min at 94 °C, 30 s at 52 °C, 1 min at 72 °C, and a final extension at 72 °C for 8 min for the ITS gene region. The PCR amplicons were purified with a FavorPrepTM PCR purification kit as per the manufacturer’s instructions. The sequencing PCR was set up using BigDye^®^ Terminatorv3.1 Cycle Sequencing Kit as per the manufacturer’s instructions. The sequencing PCR reaction of 20 μL included 4 μL of 5× sequencing buffer, 2 μL of BigDyeTM Terminator premix, 4 μL of primer ITS 5 (5 pmol), and 4 μL of the purified amplicon and H_2_O (Sterile Ultra-Pure Water, Sigma Aldrich, Bengaluru, Karnataka, India), with the volume achieving 20 μL. Thermal cycling conditions consisted of an initial denaturing at 96 °C for 3 min, followed by 30 cycles of 94 °C for 10 s, 50 °C for 40 s, and 60 °C for 4 min. The BigDye^®^ terminators and salts were removed from using The BigDye Xterminator^®^ Purification Kit (Thermo Fisher Scientific, Waltham, MA, USA) as per the manufacturer’s instructions. The purified sequencing products were transferred into a 96-well microplate. The sequence was elucidated using the Applied Biosystems SeqStudio Genetic Analyzer (Applied Biosystems, Foster City, CA, USA). The sequence was searched against rRNA/ITS databases containing ITS sequences of fungi type and reference material to reach identity. However, the β-tubulin gene amplification was carried out with the help of forward primer BT5 and reverse primer BT6 according to the reported PCR conditions [29].

### 2.9. Evolutionary Relationships of Taxa

The evolutionary history was inferred using the neighbor-joining method [30]. The optimal tree is shown. The percentage of replicate trees where the associated taxa clustered together in the bootstrap test (1000 replicates) is shown next to the branches [31]. The tree is drawn to scale, with branch lengths in the same units as those of the evolutionary distances used to infer the phylogenetic tree. The evolutionary distances were computed using the Maximum Composite Likelihood method [32] and are in the units of the number of base substitutions per site. The proportion of sites where at least 1 unambiguous base is present in at least 1 sequence for each descendent clade is shown next to each internal node in the tree. This analysis involved 34 nucleotide sequences. All ambiguous positions were removed for each sequence pair (pairwise deletion option). There were a total of 612 positions in the final dataset. Evolutionary analyses were conducted in MEGA11 [33].

### 2.10. Bioefficacy of Different Treatments Against the Test Pathogen

A single mycelial disc of 5 mm diameter of *P. aphanidermatum* was placed aseptically at one end of the Petri plate. After 2 days of growth of *P. aphanidermatum*, a 5 mm diameter mycelial disc (taken from the margin of a 2-day-old culture) of the *T. harzianum* was placed at the opposite end of the same plate. This was performed to adjust the growth rates of the pathogen, i.e., *P. aphanidermatum* and antagonist, i.e., *T. harzianum*, so that they come in contact near the centre of the plate simultaneously. The plates were then incubated at 28 ± 1 °C in a B.O.D. incubator. After contact with the pathogen and antagonist, the overgrowth by antagonist/pathogen over the pathogen/antagonist was recorded. The degree of interaction was rated according to the scale described below.
Class 1The *T. harzianum* completely overgrew the *P. aphanidermatum* (100% overlap)Class 2The *T. harzianum* overgrew at least ^2^/_3_ of *P. aphanidermatum* (75% overlap)Class 3The *T. harzianum* overgrew at least ½ of *P. aphanidermatum* (75% overlap)Class 4The *P. aphanidermatum* and the *T. harzianum* locked at the point of contactClass 5The *P. aphanidermatum* overgrew the *T. harzianum.*The interaction was observed regularly until 9 days after contact.

The in vitro antagonistic effect of *P. fluorescens* against the fungal pathogen *P. aphanidermatum* was assessed by allowing both organisms to grow using 20 mL of King’s B+ PDA medium (1:1) by dual culture techniques [34]. Pure and fresh cultures of *P. fluorescens* and *P. aphanidermatum* were cultured for 4–5 days, respectively. For the antagonistic study, a mycelial disc of a 5 mm diameter agar plug containing *P. aphanidermatum* hyphae was placed in the centre of a Petri dish containing dual media under aseptic conditions. *P. fluorescens* was streaked in circular form, maintaining a 3 cm distance from the test pathogen [34]. For control, Petri plates containing the pathogen *P. aphanidermatum* were considered. The Petri plates were incubated at 28  ±  2 °C for 6 days, with 3 replications and the observations were recorded. Percent growth inhibition of *P. aphanidermatum* by *P. fluorescens* was calculated [35].

The PDA plates containing the COC at 1500 ppm and 2000 ppm were inoculated aseptically with the isolated pathogen by transferring a 5 mm diameter agar disc of the 7-day-old culture to the centre of the Petri dish. Three replications were maintained for each treatment. The basal medium (PDA) is maintained as a control without any treatment. All the inoculated Petri dishes were incubated at 28  ±  2 °C. The radial growth of the *P. aphanidermatum* in the treated plates was measured in all treatments when the pathogen growth touched the periphery in the control Petri dishes. The percent inhibition (I) of fungal growth was estimated by using the formula [35].

(5) $$I=\frac{Colony\;diameter\;in\;control-Colony\;diameter\;in\;treatment}{Colony\;diameter\;in\;control}\times 100$$

### 2.11. Pathogenicity Test

A pathogenicity test was conducted under in vitro conditions to confirm the soft rot of ginger caused by *P. aphanidermatum*. To test the pathogenicity of *P. aphanidermatum* on ginger, 3–5 cm disease-free ginger rhizome seeds were sown in pots (1 rhizome seed having 20 cm diameter) containing non-infested soil. A month-old plant was inoculated with *P. aphanidermatum* by mixing the top soil with 100 g inoculum prepared by growing the pathogen in sand-maize meal medium (9:1) for 10 days. Control or untreated pots were also maintained against inoculated pots. Morphological characterisation of isolated fungi was performed under a microscope and structures like fungal mycelium, sporangia, oogonia, antheridium, etc., were recorded. The data on disease severity were recorded by using a 0–5 rating scale (Table 2).

### 2.12. Rhizome Yield Analysis

The rhizome yield was recorded treatment-wise and converted into hectares. The yield was recorded at its physiological maturity.

### 2.13. Rhizome Yield Loss Estimation

The relative rhizome yield loss (RRYL) due to soft rot disease was calculated using the following formula:

(6) $$RRYL=\frac{Yield\;in\;protected\;plot-Yield\;in\;unprotected\;plot}{Yield\;in\;protected\;plot}\times 100$$

### 2.14. Economic Appraisal

Economic appraisal, *viz*., cost of cultivation (Rs/ha), gross income (Rs.), additional income/ha over control (Rs.), net income (Rs.) and benefit–cost ratio (BCR) were calculated. BCR was calculated by dividing the total net income by the cost of cultivation.

### 2.15. Statistical Analysis

Rhizome germination (%), disease incidence (%), PDI (%), CODEX (%), and yield data (t/ha) were recorded separately. However, treatment-wise, rhizome yield data were recorded at its physiological maturity stage. The disease incidence (%) and PDI (%), data were arc-sine transformed before analysis. Graphical studies of different soil chemical parameters, disease incidence and PDI have been illustrated, and data on rhizome germination, plant height, disease incidence, PDI, and soil chemical parameters were analysed using version 16 of SPSS (Statistical Package for the Social Sciences) Inc., (Chicago, IL, USA) to conduct Duncan’s multiple range test (DMRT) and Pearson’s correlation. The data collected were subjected to statistical analysis by comparing the means of all parameters through “F” test, and the differences exhibited by the treatments were tested for their significance [36]. Each treatment Standard error of means [S.Em (±)] and Least Significant difference (LSD) at 0.05 probabilities (*p* = 0.05) were also calculated.

## 3. Results

### 3.1. Disease Symptoms

**Figure 2:**
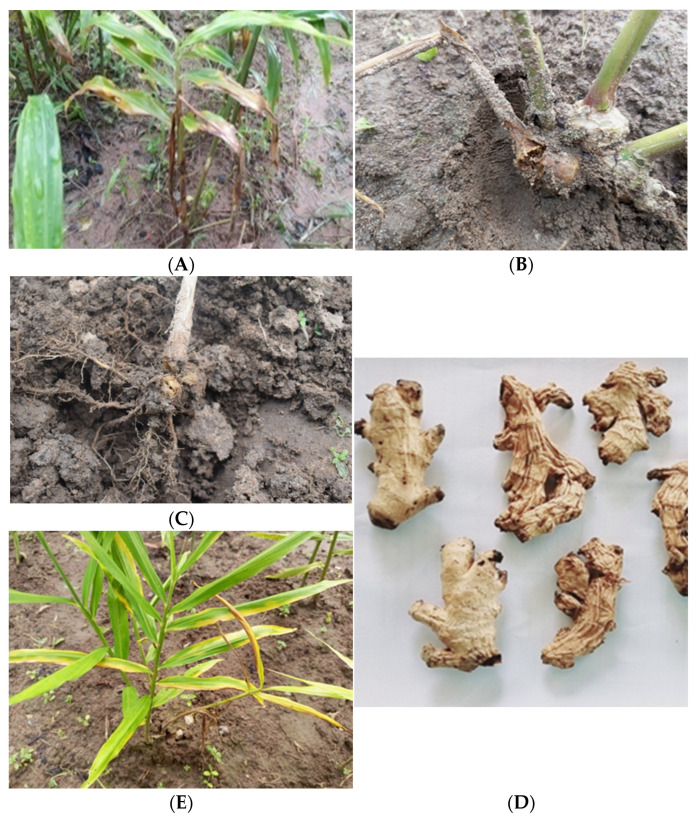
Characteristic symptoms of soft rot of ginger: (**A**) Infected plants showing yellowing symptoms. (**B**) The collar region of pseudostems showing water-soaked symptoms. (**C**) The rotting of infected rhizomes. (**D**) Dried infected rhizomes. (**E**) Yellowing symptoms.

The pathogen infects the ginger crop throughout the cropping season and shows light yellowing of leaf tips. The yellowing symptoms start from the lower to upper leaves (Figure 2A), and the infected collar region of pseudostems becomes water-soaked (Figure 2B). The infected rhizomes become rotten, soft, and brown-discoloured, and develop a distinct foul smell (Figure 2C,D). As the disease progresses, the whole plant shows yellowing symptoms followed by withering and drying (Figure 2E). The symptomatic plants showed stunted growth with a distinct foul smell.

### 3.2. Identification of the Pathogen

**Figure 3:**
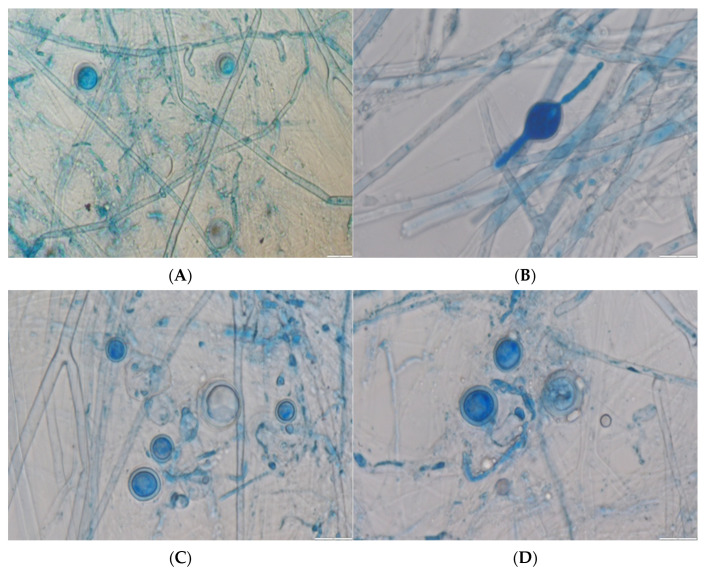
Microscopic view of *P. aphanidermatum*: (**A**) Cenocytic hypha. (**B**) Outgrowing papillae. (**C**) Oogonium. (**D**) Antheridium. All organs observed under light microscope at 40× to 400× magnification.

**Figure 4:**
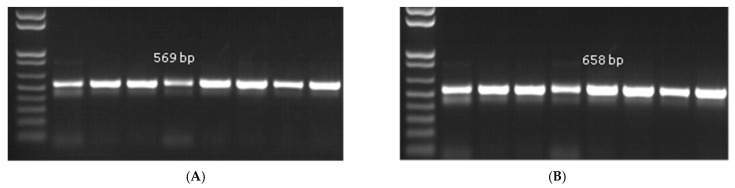
Agarose gel electrophoresis (1%) image of PCR amplification (**A**). ITS amplicon: 569 bp band (**B**). β-tubulin amplicon: 658 bp band), Marker-100 bp.

*Pythium aphanidermatum* was isolated and identified from the diseased tissues of ginger. All the eight samples analysed, through both morphological and molecular identification methods, resulted in the same findings. The isolates (R. G. 1) were sent for further molecular identification based on sequencing with ITS and β-tubulin. The morphological and microscopic identifications showed that the fungal colonies on PDA and OMA at 25 ± 2 °C after 07 days were fast-growing, half-white coenocytic, guttulate, smooth-walled, hyaline, 2.66–9.58 μm, distinctive hyphal swellings (Figure 3A). Sporangia were considered globose to subglobose, with stalks produced from lateral hyphae, measuring 10.85–17.68 × 10.40–14.06 μm. Papillae developed in a discharge tube (Figure 3B). Oogonia were abundantly produced, being considered globose to subglobose, thick-walled, and multilayered, measuring 11.41–24.74 × 11.25–24.80 μm (Figure 3C). Antheridia developed from the same stalk, being considered monocline to dicline, curved, and clavate in shape (Figure 3D). Amplification of the ITS genes and their sequence were completed using PCR. The available sequences in GenBank were used to compare the sequences using the BLAST programme (http://www.ncbi.nlm.nih.Gov/BLAST, accessed on 30 January 2025). Molecular characterisation revealed the identity of the isolates as *P. aphanidermatum* (Figure 4A,B). Furthermore, it was observed that the polygenetic relationship of the fungal isolate is very close to the type strains of the *Pythium* genus (Supplementary Figure S1). The culture of the oomycetes has been deposited with the National Fungal Culture Collection of India at Agharkar Research Institute, Pune, India with accession number NFCCI 5832, and the sequence was deposited at GenBank of NCBI with accession number PQ772614 for ITS and PV399882 for *β-tubulin*. A BLAST search showed 100% identity with *P. aphanidermatum* strain CBS 118.80 (GenBank accession numbers AY598622.1 for ITS; KJ595472.1 for *β-tubulin*).

### 3.3. Pathogenicity Test

**Figure 5:**
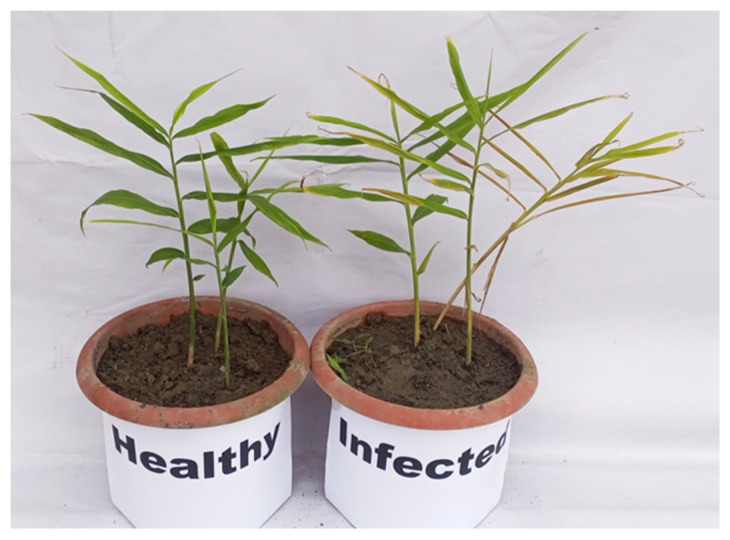
Pathogenicity test of the *P*. *aphanidermatum* isolate on ginger.

Pots inoculated with *P. aphanidermatum* showed symptoms of yellowing leaves and wilting, whereas the uninoculated pot showed healthy plants, as depicted in Figure 5. Water soaked and brown lesions were observed on the rhizome. These lesions then enlarge and coalesce, leading to stem rot and plant collapse.

### 3.4. Rhizome Germination (%)

**Figure 6:**
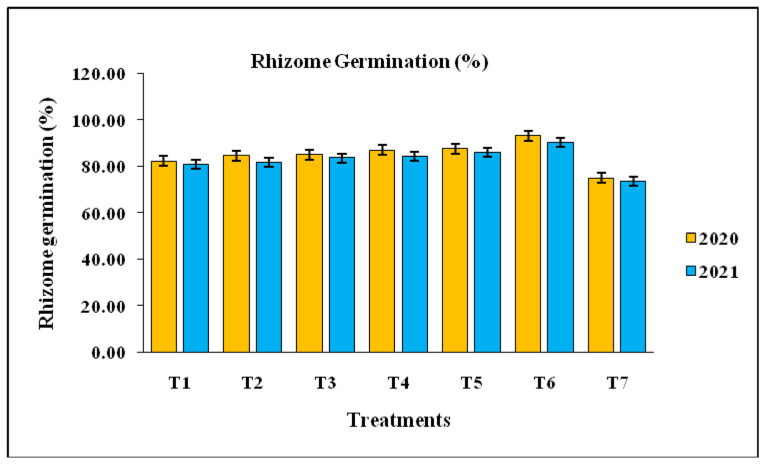
Effects of different treatments on rhizome germination of ginger. Error bar represents standard error. T1, *T. harzianum* at the rate 10 g/kg of rhizomes followed by air drying for 24 h at a normal room temperature; T2, *P. fluorescens* at the rate 10 g/kg of rhizomes followed by air drying for 24 h at a normal room temperature; T3, copper oxychloride (COC) at the rate 0.3% (3 g/L) for 30 min and air dried before sowing; T4, *P. fluorescens* at the rate 10 g/kg of rhizomes + soil application of *P. fluorescens* enriched FYM (3 kg of *P. fluorescens* mixed with 100 kg FYM at 10–15 days before sowing) + soil drenching with *P. fluorescens* at the rate 10 kg/ha; T5, *T. harzianum* (10 g/kg of rhizomes) + soil application of *T. harzianum*-enriched well-decomposed farm yard manure (3 kg of *T. harzianum* mixed with 100 kg FYM at 10–15 days before sowing) + soil drenching with *T. harzianum* at the rate 10 kg/ha; T6, COC at the rate 3 g/L and streptomycin sulphate 90% + tetracycline hydrochloride 10% WP at the rate 0.2 g/L for 45 min followed by shade drying and two soil drenching with COC at the rate 3 g/L at 60 and 90 days after planting (standard check); T7, control.

Data revealed that all the treatments tested were effective against soft rot disease and showed significantly increased rhizome germination over the untreated check (Figure 6). Among the treatments, rhizome treatment with COC at the rate of 3 g/L and streptomycin sulphate 90% + tetracycline hydrochloride 10% WP at the rate of 0.2 g/L for 45 min, followed by shade drying and two soil drenching with COC at the rate 3 g/L at 60 and 90 days after planting recorded highest percent rhizome germination (93.33% and 90.33%, respectively) as compared to control (75.00% and 73.67%, respectively). This was followed by rhizome treatment with *T. harzianum* (10 g/kg of rhizomes) + soil application of *T. harzianum*-enriched well-decomposed farm yard manure (3 kg of *T. harzianum* mixed with 100 kg FYM at 10–15 days before sowing) + soil drenching with *T. harzianum* at the rate 10 kg/ha (87.67% and 86.00%, respectively). Both treatments were statistically significantly different from each other in 2020; however, they were statistically at par in 2021. A perusal of the pooled data (Table 3) revealed that plant height was highest (91.83%) with the application of COC at the rate 3 g/L and streptomycin sulphate 90% + tetracycline hydrochloride 10% WP at the rate of 0.2 g/L for 45 min followed by shade drying and soil drenching twice with COC at the rate 3 g/L at 60 and 90 days after planting, followed by the application of *T. harzianum* (10 g/kg of rhizomes) + soil application of *T. harzianum*-enriched well-decomposed farm yard manure (3 kg of *T. harzianum* mixed with 100 kg FYM at 10–15 days before sowing) + soil drenching with *T. harzianum* at the rate 10 kg/ha (86.83%) compared to control (74.33%).

### 3.5. Plant Height

**Figure 7:**
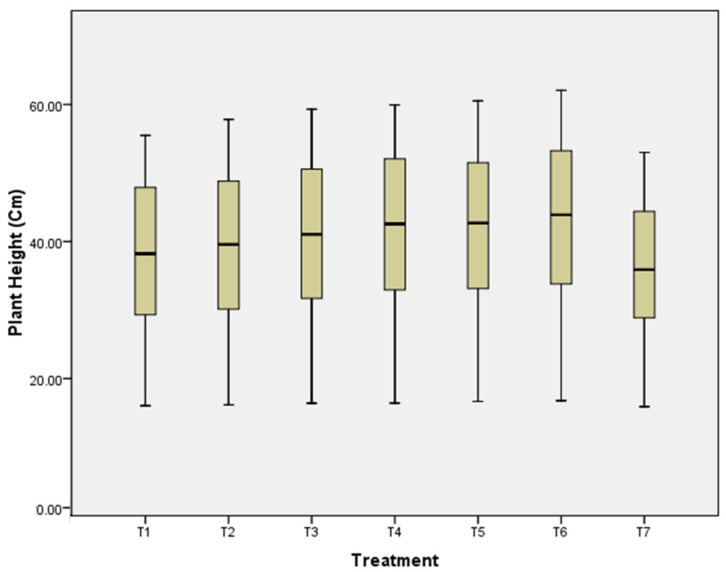
Effects of different treatments on plant height.

Among the treatments evaluated, the average (pooled) highest plant height of 16.85 cm, 33.95 cm, 43.89 cm, 52.92 cm, 61.39 cm (average plant height, 41.80 cm) and 16.72 cm, 33.20 cm, 42.70 cm, 51.37 cm, 60.13 cm (average plant height, 40.82 cm) was recorded with rhizome treatment with COC at the rate 3 g/L and Streptomycin sulphate 90% + tetracycline hydrochloride 10% WP at the rate of 0.2 g/L for 45 min, followed by shade drying and two soil drenching with COC at the rate of 3 g/L at 60 and 90 days after planting and rhizome treatment with *T. harzianum* (10 g/kg of rhizomes) + soil application of *T. harzianum*-enriched well-decomposed farm yard manure (3 kg of *T. harzianum* mixed with 100 kg FYM at 10–15 days before sowing) + soil drenching with *T. harzianum* at the rate 10 kg/ha at 32, 62, 92, 122 and 152 days after sowing (DAS), respectively, during 2020 and 2021 as compared to controls (Figure 7) which were statistically at par with each other. The pooled data (Table 3) of plant height at 152 DAS indicated that the maximum increase in plant height (61.38 cm) was recorded with rhizome treatment with COC at the rate 3 g/L and Streptomycin sulphate 90% + tetracycline hydrochloride 10% WP at the rate of 0.2 g/L for 45 min, followed by shade drying and soil drenching twice with COC at the rate of 3 g/L, at 60 and 90 days after planting, followed by rhizome treatment with *T. harzianum* (10 g/kg of rhizomes) + soil application of *T. harzianum*-enriched with well-decomposed farm yard manure (3 kg of *T. harzianum* mixed with 100 kg FYM at 10–15 days before sowing) + soil drenching with *T. harzianum* (60.13 cm), compared to control (52.77 cm). Both treatments were statistically significantly different from each other.

### 3.6. Disease Incidence

**Figure 8:**
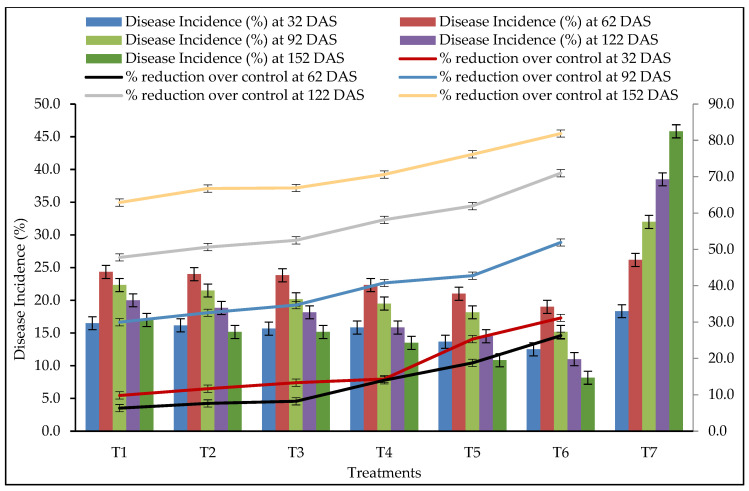
Effects of different treatments on disease incidence (pooled data from 2020 and 2021). DAS: days after sowing, error bar represents standard error.

Different treatments significantly influenced soft rot disease incidence. Rhizome treatment with *T. harzianum* (10 g/kg of rhizomes) + soil application of *T. harzianum*-enriched well-decomposed farm yard manure (3 kg of *T. harzianum* mixed with 100 kg FYM at 10–15 days before sowing) + soil drenching with *T. harzianum* at the rate 10 kg/ha found average (pooled) smallest disease incidence of 13.67%, 21.00%, 18.17%, 14.50%, 10.83% with average disease reduction of 25.33%, 18.77%, 42.77%, 61.94%, 76.17% at 32, 62, 92, 122 and 152 DAS, respectively, after treatment, as compared to the control (Figure 8). The difference between the T5 and T6 treatments was statistically significant (*p* < 0.05) at 32,122, 152 DAS and at par at 62, 92 DAS. This may be due to the secretion of secondary metabolites by *T. harzianum*, which inhibit zoospore germination, germ tube elongation and mycelial growth of *P. aphanidermatum*.

### 3.7. Disease Severity

**Figure 9:**
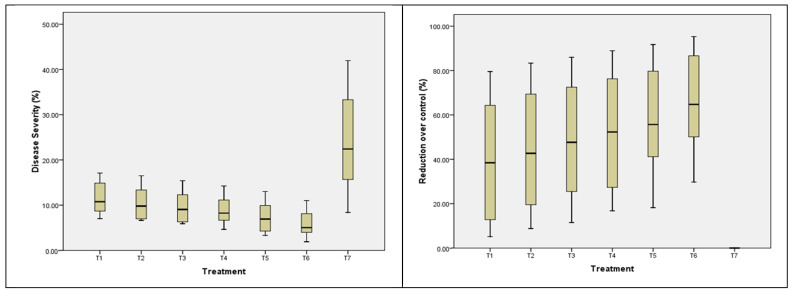
Effects of different treatments on disease severity and percent reduction over control (pooled data from 2020 and 2021).

Data also revealed that all the treatments significantly decreased the disease severity index at 32, 62, 92, 122 and 152 DAS. The average (pooled) disease severity index was recorded in the range of 4.90% to 10.21%, 10.89% to 17.63%, 7.86% to 22.70%, 4.22% to 29.11% and 1.93% to 38.12% at 32, 62, 92, 122 and 152 DAS, respectively (Figure 9). However, the average percent reduction over control was in the range of 79.43% to 94.89%. The minimum average disease severity index was recorded with rhizome treatment with COC at 3 g/L and streptomycin sulphate 90% + tetracycline hydrochloride 10% WP at the rate of 0.2 g/L for 45 min, followed by shade drying and planting and two soil drenching with COC at the rate of 3 g/L at 60 and 90 days after planting (5.84 and 6.08%) with maximum average percent reduction over control (63.52 and 70.10%) followed by rhizome treatment with *T. harzianum* (10 g/kg of rhizomes) + soil application of *T. harzianum*-enriched well-decomposed farm yard manure (3 kg of *T. harzianum* mixed with 100 kg FYM at 10–15 days before sowing) + soil drenching with *T. harzianum* at the rate 10 kg/ha (disease severity: 7.25 and 8.04%; cent reduction over control: 56.83 and 61.47%) during 2020 and 2021, respectively. The findings suggest that treatment T5 was statistically at par with treatment T6 at 32, 92, 122 DAS and significant at 62, 152 DAS. Two-year pooled analysis of data (Table 3) concluded that smallest disease severity indexes of 4.90%, 10.89%, 7.86%, 4.22% and 1.93% were recorded with treatment T6, followed by T5, which exhibited disease severity indexes of 5.67%, 12.92%, 9.90%, 6.34% and 3.39%, as compared to control at 32, 62, 92, 122 and 152 DAS, respectively. Both the treatments were statistically significant at 62, 92, 122 DAS and at par at 32, 152 DAS. This may be due to the mycoparasitic activity of *T. harzianum* and the production of β13-Endoglucanase, Subtilisin-like protease, and chitinase to break down the cell walls of pathogens.

### 3.8. Soil Chemical Properties

The correlation indicated a negative relationship between disease severity and all indicators, except for electrical conductivity, which is also negatively correlated with soil organic carbon (SOC) and NPK, presented in Table 4. The relation between disease severity with SOC, nitrogen, and phosphorus exhibits a strong negative correlation relative to potassium, which may be attributable to defensive chemicals such as phenols, produced due to potassium, which provide resistance against pests and pathogens. Potassium aids in the fortification of plant cell walls and tissues, enhancing resistance against infections and pests. The strong positive association between SOC and primary nutrients may result from nutrients produced during the breakdown of organic materials.

**Figure 10:**
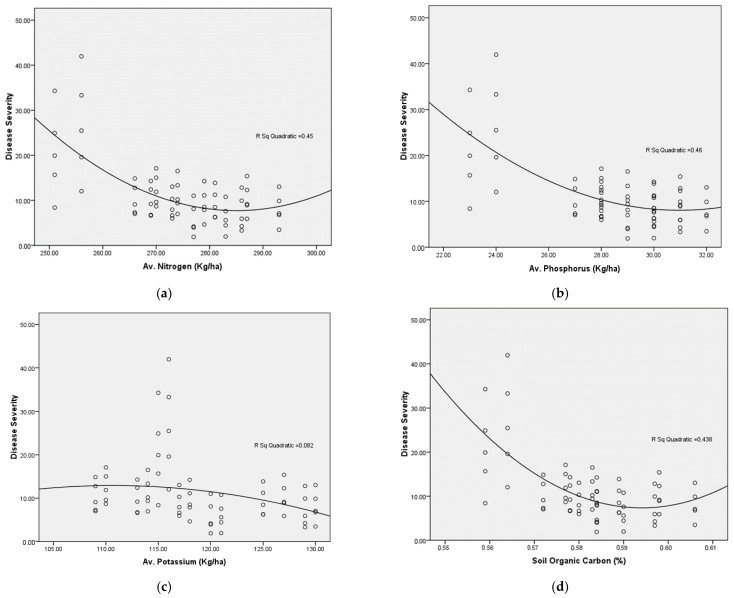
The quadratic curve fitting between disease severity and soil chemical parameters. (**a**) Relationship between disease severity and soil available nitrogen. (**b**) Relationship between disease severity and soil available phosphorus. (**c**) Relationship between disease severity and soil available potassium. (**d**) Relationship between disease severity and soil organic carbon.

Figure 10a–d illustrates the quadratic curve fitting between disease severity and soil-available nitrogen, phosphate, potassium, and organic carbon. The availability of nitrogen, phosphorus, and soil organic carbon first decreases and subsequently increases disease severity, whereas potassium exhibits a smaller boost, but mainly decreases disease severity with higher dosages. While nitrogen and phosphorus inputs augment plant defences, they concurrently elevate the availability of nitrogen and phosphorus compounds for pathogen exploitation, and excessive application of these fertilisers has been shown to exacerbate disease development. Increased N and P supplies may enhance disease susceptibility by altering the canopy structure, which may create an environment conducive to pathogen development.

**Figure 11:**
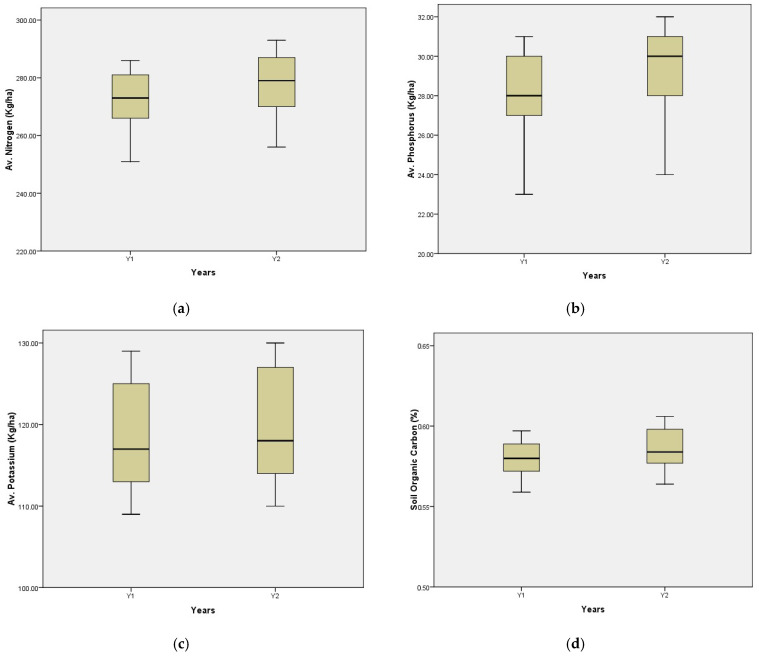
The box-and-whisker plots of soil chemical parameters over the years. (**a**) Relationship between soil available nitrogen and years. (**b**) Relationship between soil available phosphorus and years. (**c**) Relationship between soil available potassium and years. (**d**) Relationship between soil organic carbon and years.

The box-and-whisker plot (Figure 11) illustrates a variation in soil organic carbon (d), available nitrogen (a), phosphate (b), and potassium (c) across the experimental years. However, an uneven distribution was seen in soil organic carbon, available phosphorus, and potassium, whereas available nitrogen was reasonably distributed. Data distribution is sustained, with an increasing value in the subsequent year. The median of the available phosphorus varies over the years of evaluation, maybe due to the addition of phosphorus by bio-agents.

**Figure 12:**
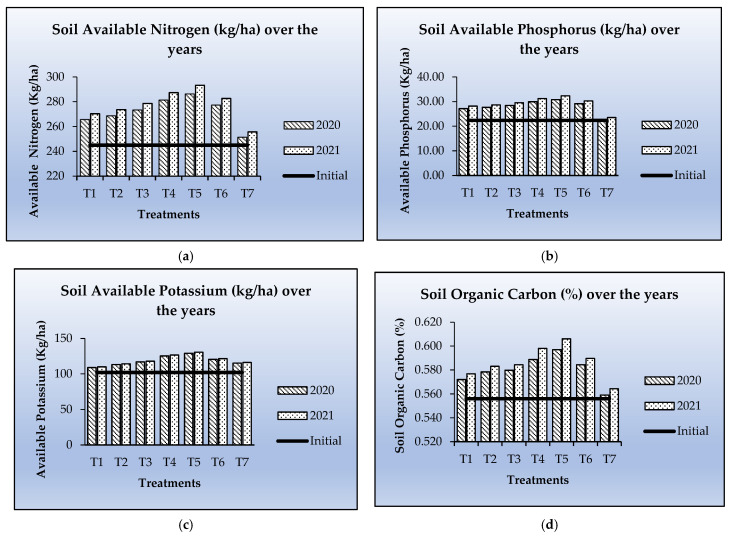
Soil chemical parameters over the years.

Figure 12a–d portrays the impact of treatment on available nutrients and soil organic carbon (SOC). The data unequivocally illustrates that nutrient content was increased across all treatments from the starting levels of available nutrients and soil organic carbon (SOC). Nonetheless, a slight increase in the available phosphorus and potassium concentrations was seen across the treatments, likely attributable to phosphorus immobilisation and inadequate release of exchangeable potassium from the soil colloids in the region’s acidic soils.

### 3.9. Bioefficacy of Different Treatments Against the Test Pathogen

**Figure 13:**
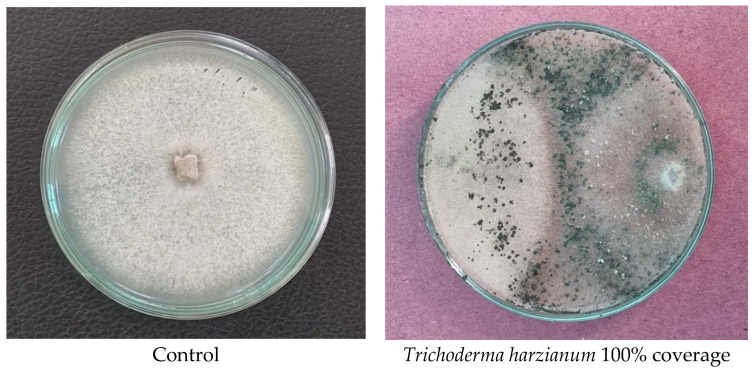
Bioefficacy of *T. harzianum* against *P. aphanidermatum*.

Evaluations of bio-agents and COC against *P. aphanidermatum* show that the percent inhibition of mycelia growth (%) for COC was 90.7% at 1500 ppm and 94.2% at 2000 ppm. Although the percent inhibition of mycelia growth was 65.5 for *T. harzianum* and 60.2% for *P. fluorescens* (Table 5), *T. harzianum* completely parasitizes *P. aphanidermatum* after 9 days of growth (Figure 13).

### 3.10. CODEX

Present data (Table 6) revealed that the lowest CODEX value recorded was in the rhizome treatment with Copper oxychloride at the rate 3 g/L + streptomycin, at the rate 0.2 g/L, for 45 min, followed by shade drying and planting and soil drenching twice with COC at the rate 3 g/L, at 60 and 90 days after planting (0.60%), followed by rhizome treatment with *T. harzianum* at the rate 5 g/kg of rhizomes + soil application of 2.5 kg of *T. harzianum* mixed with 50 kg FYM 10–15 days before sowing + foliar application of *P. fluorescens* at the rate 5 g/L of water for every 15 days interval (0.88%), as compared to untreated control (5.21%).

### 3.11. Rhizome Yield

Rhizome yield data revealed that the efficacy of different treatments against soft rot disease incidence and severity and their effects on rhizome yield indicated that all the treatments were significantly superior to the control, reducing the disease incidence and severity and increasing the rhizome yield. The percentage of the yield increase over control was found to be within the range of 9.01 to 25.19 percent, with rhizome yield in the range of 7.48 t/ha to 9.36 t/ha (Table 6). The average (pooled) highest rhizome yield was obtained from rhizome treatment, with COC at 3 g/L. Streptomycin sulphate 90% + Tetracycline hydrochloride 10% WP at the rate of 0.2 g/L for 45 min, followed by shade drying and planting and two soil drenching with COC at the rate of 3 g/L at 60 and 90 days after planting (9.36 t/ha) followed by rhizome treatment with *T. harzianum* (10 g/kg of rhizomes) + soil application of *T. harzianum*-enriched well-decomposed farm yard manure (3 kg of *T. harzianum* mixed with 100 kg FYM at 10–15 days before sowing) + soil drenching with *T. harzianum* at the rate 10 kg/ha (9.10 t/ha). This was followed by rhizome treatment with *P. fluorescens* at the rate of 10 g/kg of rhizomes + soil application of *P. fluorescens* enriched FYM (3 kg of *P. fluorescens* mixed with 100 kg FYM at 10–15 days before sowing) + soil drenching with *P. fluorescens* at the rate 10 kg/ha (rhizome yield: 8.68 t/ha).

### 3.12. Economic Appraisal

All the treatments significantly reduced the severity of soft rot disease incidence, resulting in maximum rhizome yield, maximum gross and additional income over control, and significantly higher BCR. From the present investigation, the average (pooled) highest gross income, additional income and net income of Rs. 500,592/ha, Rs. 89,283/ha, Rs. 377,920/ha was recorded with rhizome treatment with *T. harzianum* (10 g/kg of rhizomes) + soil application of *T. harzianum*-enriched well-decomposed farm yard manure (3 kg of *T. harzianum* mixed with 100 kg FYM at 10–15 days before sowing) + soil drenching with *T. harzianum* at the rate 10 kg/ha after treatment standard check over control (gross income: Rs. 411,308/ha, and net income: Rs. 298,608/ha). Considering the BCR, the most economical treatment, which recorded the average (pooled) highest BCR (1:3.08), was rhizome treatment with *T. harzianum* (10 g/kg of rhizomes) + soil application of *T. harzianum*-enriched well-decomposed farm yard manure (3 kg of *T. harzianum* mixed with 100 kg FYM at 10–15 days before sowing) + soil drenching with *T. harzianum* at the rate 10 kg/ha after treatment standard check over control (1:2.65).

Therefore, rhizome rot disease management through biological control agents such as *T. harzianum*, and *P. fluorescens* are gaining more attention as a part of integrated pest management modules to reduce the negative impact of chemical pesticides. The various bioactive compounds secreted by the biological control agents act as antimicrobial activity against *P. aphanidermatum* and stimulate the growth of plants.

## 4. Discussion

Rhizome rot of ginger caused by *P. aphanidermatum* is an important destructive disease which poses a significant threat to ginger cultivation [37,38]. *Pythium* species are a group of microorganisms that resemble fungi but belong to a distinct kingdom called Stramenopila. Specifically, they are part of the family Pythiaceae, order Peronosporales, and phylum Oomycota [39]. Stramenopila has features like unique reproductive structures, biflagellate zoospores, distinctive cell structure, and thallus comprising aseptate hyphae with cell walls rich in β-glucans and cellulose, and they exhibit a diploid vegetative state. Scientists worldwide are paying close emphasis to using biological control tools to manage plant disease. Many microorganisms that fight off plant diseases, primarily fungi and bacteria, have been identified. These comprise *Trichoderma* spp., *Pseudomonas*, *Bacillus*, *Streptomyces*, mycoparasitic *Verticillium* sp., and *Lecanicillium* sp. BCA causes physiological alterations in plants, like the development of phytohormones or the production of chitinase and glucanase [40]. *Pythium* can infect seeds or roots and incite long-lasting root rots; despite these challenges, antagonistic fungi, microbes, and actinomycetes are effective against the pathogen [40,41,42].

*Trichoderma* spp. is a promising biocontrol agent that protects plants from pathogenic microorganisms by triggering the plant defence mechanism and secretion of secondary metabolites [43,44,45,46,47,48,49]. A wide range of *Pythium* species can be managed by using *Trichoderma*. The findings align with previous research [50], stating that maximum plant height was recorded with *Trichoderma* spp. + Neem extract rhizome seed treatment, followed by copper oxychloride rhizome seed treatment. Using bio-control agents such as *T. harzianum* can effectively suppress the rhizome rot of ginger [37,51]. The antagonist degrades the cell walls of pathogens and produces indole acetic acid and cyanide, phosphate solubility, and other factors that cause cellular wall degradation of *Pythium* spp. Fungal endophytes like *T. harzianum* significantly reduce the mycelium development responsible for turmeric rhizome rot infection [52]. *T. harzianum* can solubilise several inaccessible soil nutrients to available forms for plants [53] and in vitro test of antagonistic fungi like *T. viride*, *T. hamatum*, and *T. harzianum* towards *P. aphanidermatum*, *F. solani*, and *F. equiseti* have been well documented [54,55,56,57]. BCAs reduce the proliferation of *Fusarium oxysporum* and *P. aphanidermatum*, inducing yellowing and soft rot in ginger [58,59,60]. It was reported that, among the bioagents evaluated under in vitro conditions against *P. aphanidermatum*, *T. viride*, *T. koningii* and *T. harzianum* were found most effective with the highest mycelial growth inhibition of the test pathogen [61]. These results conform the earlier findings [62,63,64,65,66].

*P. fluorescens* controls pathogens by producing various secondary metabolites like pterines, indoles, alignate, siderophores, indole-3-acidic acid, pyroles, lipids or pyco compounds, phenazines, peptides antibiotics amino acids, etc. [67]. Likewise, during the present case study, *P. fluorescens* significantly reduced the disease incidence of soft rot in ginger. The result is in tune with the previously reported results [67], revealing that *P. fluorescens* is a potential bio-agent for the rhizome rot of ginger alone or as a component of integrated disease management strategies.

The soft rot disease (67%) with reduced disease incidence (4.6%) and maximum yield (10.4 t/ha) was observed with rhizome treatment by copper hydroxide and its soil drenching, followed by *T. viride* [68]. The biocontrol agent *viz*., *Trichoderma*-treated plot showed slightly lower ginger rhizome yield than fungicide *viz*., copper hydroxide. Still, being a biocontrol agent, it is environmentally friendly and improves antagonistic activities. These findings align with earlier research [51,63,68,69,70,71,72,73,74]. BCAs represent a holistic, sustainable approach to crop protection, offering environmentally friendly, cost-effective, and long-term solutions [75,76]. Spraying with 0.2 percent Metalaxyl mancozeb + 0.015 percent streptocyclin recorded minimum disease intensity (5.67%), maximum percent disease control (79.09%) and rhizome yield (17.55 t/ha) followed by *T. viride* (disease intensity: 6.9%, percent disease control: 74.55% and rhizome yield: 15.94 t/ha, respectively) [75]. Similar results were reported earlier [68,77,78]. Applying biocontrol agents as a seed treatment, soil application, and foliar application are eco-friendly and sustainable crop protection approaches. Biocontrol agents can boost plant defences, rendering the entire plant more resistant to pathogens [79].

## 5. Conclusions

Soft rot in ginger is one of the most destructive diseases wherever the crop is grown. Integrating fungal and bacterial biocontrol agents is an effective strategy for managing soil-borne plant diseases because it combines multiple mechanisms of action against pathogens. Fungal and bacterial biocontrol agents can suppress phytopathogens through competition for resources, antibiosis, and the induction of plant defence mechanisms. Even though applying BCAs to manage soft rot disease is slightly less effective than fungicides, it is a friendly and sustainable management approach. Hence, harnessing the multifaceted potential of BCAs is a holistic approach that can offer environmentally friendly, cost-effective, and long-term sustainable crop protection for sustainable agriculture.

## Figures and Tables

**Table 1:** The initial soil parameters of the experimental site.

Soil Properties	0–30 cm Depth
pH	5.01
EC (ds/m)	0.82
SOC (%)	0.556
N (kg/ha)	245
P (kg/ha)	22.4
K (kg/ha)	102
S (ppm)	22.6
Mn (ppm)	28
B (ppm)	0.86
Fe (ppm)	23.58
Cu (ppm)	0.22
Zn (ppm)	0.86

**Table 2:** The rating scale for the assessment of soft rot in ginger.

Severity Scale	Rating Grade (%)	Level of Resistance/Susceptibility
0	0	No infection on rhizome
1	0.1–5.0	0.1–5.0% rotting of rhizome
2	5.1–15.0	5.1–15.0% rotting of rhizome
3	15.1–30.0	15.1–30.0% rotting of rhizome
4	30.1–60.0	30.1–60.0% rotting or rhizome
5	More than 60	More than 60% rotting of rhizome.

**Table 3:** Effect of different treatments on rhizome germination, plant height, disease incidence and PDI.

Treatments	Rhizome Germination (%)	Plant Height (cm) at 152 DAS	Disease Incidence (%)					PDI				
			32 DAS	62 DAS	92 DAS	122 DAS	152 DAS	32 DAS	62 DAS	92 DAS	122 DAS	152 DAS
T1	81.67 ^d^	55.10 ^e^	16.50 ^b^	24.33 ^b^	22.33 ^b^	19.33 ^b^	16.50 ^b^	8.47 ^b^	15.98 ^ab^	13.91 ^b^	10.50 ^b^	7.85 ^b^
T2	83.17 ^cd^	57.33 ^d^	16.17 ^b^	24.00 ^b^	21.50 ^b^	18.50 ^b^	15.00 ^bc^	8.06 ^b^	15.40 ^b^	12.87 ^bc^	9.71 ^bc^	6.81 ^bc^
T3	85.67 ^bc^	59.93 ^b^	15.83 ^b^	22.33 ^cd^	18.83 ^cd^	15.83 ^cd^	13.33 ^c^	7.59 ^b^	14.63 ^cd^	11.77 ^de^	8.82 ^cd^	6.10 ^d^
T4	84.33 ^bcd^	58.85 ^c^	15.67 ^b^	23.83 ^bc^	20.83 ^bc^	17.83 ^bc^	15.17 ^bc^	7.59 ^b^	13.64 ^bc^	10.72 ^cd^	7.92 ^bc^	5.32 ^cd^
T5	86.83 ^b^	60.13 ^b^	13.67 ^c^	21.00 ^d^	18.17 ^d^	14.50 ^d^	10.83 ^d^	5.67 ^c^	12.92 ^d^	9.90 ^e^	6.34 ^d^	3.39 ^e^
T6	91.83 ^a^	61.38 ^a^	12.50 ^c^	19.00 ^e^	15.17 ^e^	11.00 ^e^	8.17 ^e^	4.90 ^c^	10.89 ^e^	7.86 ^f^	4.22 ^e^	1.93 ^f^
T7	74.33 ^e^	52.77 ^f^	18.33 ^c^	26.00 ^a^	32.00 ^a^	38.50 ^a^	46.83 ^a^	10.21 ^a^	17.63 ^a^	22.70 ^a^	29.11 ^a^	38.12 ^a^
LSD (*p* ≤ 0.05)	2.86	1.07	1.43	1.59	1.69	1.84	1.88	1.57	1.32	1.62	2.04	1.84

Means followed by the same letters (a–f) in a column are not significant at 0.05.

**Table 4:** The Pearson correlation coefficient analysis between soil chemical parameters and disease severity.

	pH	Electrical Conductivity (dS/m)	Soil Organic Carbon (%)	Av. Nitrogen (Kg/ha)	Av. Phosphorus (Kg/ha)	Av. Potassium (Kg/ha)	Disease Severity
pH	1	−0.991 **	0.981 **	0.966 **	0.926 **	0.886 **	−0.522 **
Electrical Conductivity (dS/m)		1	−0.976 **	−0.958 **	−0.907 **	−0.898 **	0.508 **
Soil Organic carbon (%)			1	0.989 **	0.966 **	0.805 **	−0.555 **
Av. Nitrogen (Kg/ha)				1	0.986 **	0.760 **	−0.600 **
Av. Phosphorus (Kg/ha)					1	0.669 **	−0.631 **
Av. Potassium (Kg/ha)						1	−0.273 *
Disease Severity							1

** Correlation is significant at the 0.01 level (2-tailed). * Correlation is significant at the 0.05 level (2-tailed).

**Table 5:** Evaluations of bio-agents and COC against *P. aphanidermatum*.

Sl No.	Bio Agent/Fungicide (COC)	Percent Inhibition of Mycelial Growth (%)
1	*Trichoderma harzianum*	65.5
3	*Pseudomonas fluorescens*	60.2
4	COC 1500 ppm	90.7
5	COC 2000 ppm	94.2

**Table 6:** Economics of different treatments for managing rhizome rot disease in ginger (pooled data of 2020 and 2021).

Treatments	Gross Cost of Cultivation (Rs/ha)				CODEX (%)	Rhizome Yield (t/ha) *	% Increase over Control	RRYL	Gross Income ** (Rs.)	Additional Income/ha over Control	Net Profit (Rs.)	B:C Ratio
	Cost of Cultivation	Treatments	Labour Charges	Total (Rs.)								
T1	112,700	3735	1000	117,435	1.81	8.15	9.01	8.26	448,158	36,850	330,723	1:2.82
T2	112,700	3675	1000	117,375	1.60	8.35	11.69	10.46	459,250	47,942	341,875	1:2.91
T3	112,700	3870	1000	117,570	1.45	8.55	14.41	12.57	469,975	58,667	352,405	1:3.00
T4	112,700	6860	3000	122,560	1.20	8.68	15.98	13.75	477,583	66,275	355,023	1:2.90
T5	112,700	6972	3000	122,672	0.89	9.10	21.85	17.91	500,592	89,283	377,920	1:3.08
T6	112,700	10,020	3000	125,720	0.55	9.36	25.19	20.10	514,525	103,217	388,805	1:3.09
T7	-	-	-	112,700	6.76	7.48	-	-	411,308	-	298,608	1:2.65
S.Em. (±)	-	-	-	-	-	0.48	-	-	-	-	-	-
C.D. (*p *= 0.05)	-	-	-	-	-	1.47	-	-	-	-	-	-
												
												

* Average of replications, ** selling rates of ginger @ Rs. 55,000/t.

## Data Availability

The data will be made available on request.

## Supplementary Materials

The following supporting information can be downloaded at: https://www.mdpi.com/article/10.3390/pathogens14060544/s1, Figure S1: Phylogenetic analysis by maximum composite likelihood method based on neighbor-joining model using sequences of ITS, and β-tubulin of *P. aphanidermatum* strains. Evolutionary analyses were conducted using MEGA11 software, 1000 bootstrap replicates.
