# Supplementary material for: Identification, Detection, and Management of Soft Rot Disease of Ginger in the Eastern Himalayan Region of India
Source: Pathogens. 2025 May 29;14(6):544. doi: 10.3390/pathogens14060544 (PMC12195653; doi:10.3390/pathogens14060544)
Supplement: Supplementary file 1 [file pathogens-14-00544-s001.zip › pathogens-3583494-supplementary.pdf]

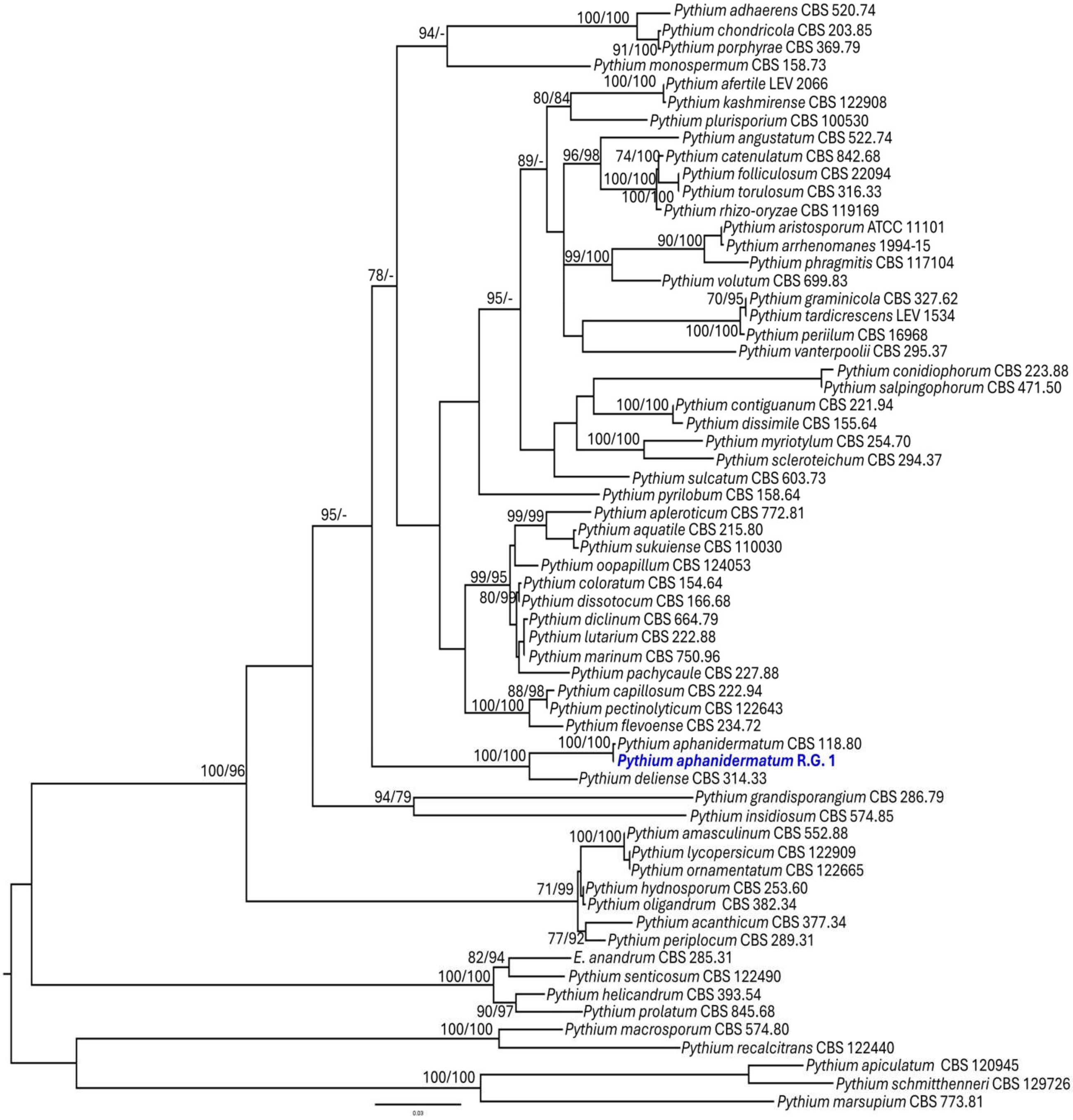

Figure S1: Phylogenetic analysis by maximum composite likelihood method based on neigh-bor-joining model using sequences of ITS, and β-tubulin of P. aphanidermatum strains. Evolutionary analyses were conducted using MEGA11 software, 1000 bootstrap replicates.
